# Cyclical Patterns of Hand, Foot and Mouth Disease Caused by Enterovirus A71 in Malaysia

**DOI:** 10.1371/journal.pntd.0004562

**Published:** 2016-03-24

**Authors:** NMN NikNadia, I-Ching Sam, Sanjay Rampal, WMZ WanNorAmalina, Ghazali NurAtifah, Khebir Verasahib, Chia Ching Ong, MohdAidinniza MohdAdib, Yoke Fun Chan

**Affiliations:** 1 Department of Medical Microbiology, Faculty of Medicine, University of Malaya, Malaysia; 2 Department of Social and Preventive Medicine, Julius Centre University of Malaya, Faculty of Medicine, University of Malaya, Malaysia; 3 National Public Health Laboratory, Ministry of Health, Selangor, Malaysia; 4 Kepong Health Office, Ministry of Health, Kuala Lumpur, Malaysia; 5 Zoonosis Sector, Disease Control Division, Ministry of Health, Malaysia; U.S. Naval Medical Research Unit No. 2, INDONESIA

## Abstract

Enterovirus A71 (EV-A71) is an important emerging pathogen causing large epidemics of hand, foot and mouth disease (HFMD) in children. In Malaysia, since the first EV-A71 epidemic in 1997, recurrent cyclical epidemics have occurred every 2–3 years for reasons that remain unclear. We hypothesize that this cyclical pattern is due to changes in population immunity in children (measured as seroprevalence). Neutralizing antibody titers against EV-A71 were measured in 2,141 residual serum samples collected from children ≤12 years old between 1995 and 2012 to determine the seroprevalence of EV-A71. Reported national HFMD incidence was highest in children <2 years, and decreased with age; in support of this, EV-A71 seroprevalence was significantly associated with age, indicating greater susceptibility in younger children. EV-A71 epidemics are also characterized by peaks of increased genetic diversity, often with genotype changes. Cross-sectional time series analysis was used to model the association between EV-A71 epidemic periods and EV-A71 seroprevalence adjusting for age and climatic variables (temperature, rainfall, rain days and ultraviolet radiance). A 10% increase in absolute monthly EV-A71 seroprevalence was associated with a 45% higher odds of an epidemic (adjusted odds ratio, aOR1.45; 95% CI 1.24–1.69; *P*<0.001). Every 10% decrease in seroprevalence between preceding and current months was associated with a 16% higher odds of an epidemic (aOR = 1.16; CI 1.01–1.34 *P*<0.034). In summary, the 2–3 year cyclical pattern of EV-A71 epidemics in Malaysia is mainly due to the fall of population immunity accompanying the accumulation of susceptible children between epidemics. This study will impact the future planning, timing and target populations for vaccine programs.

## Author Summary

Enterovirus A71 (EV-A71) is a major cause of hand, foot, and mouth disease (HFMD) in children. Since the first outbreak in Malaysia in 1997, EV-A71 epidemics have occurred every 2–3 years, in 2000, 2003, 2006, 2008/2009, and 2012. As the reasons for this cyclical pattern are not known, we hypothesize that it is due to changes in population immunity in children. In this study, we measured the EV-A71 neutralizing antibody prevalence in serum collected from children ≤12 years old between 1995 and 2012, covering 18 years and 6 epidemics. HFMD incidence was highest in children <2 years, and seroprevalence increased with age, and was higher during epidemics compared to non-epidemic periods. Peaks in EV-A71 genetic diversity coincided with reported EV-A71 epidemics. Decreases in EV-A71 seroprevalence over time were significantly associated with subsequent epidemic periods. This suggests that epidemics lead to high levels of population seroprevalence; but during the 2–3 years between epidemics, the population of young children with no immunity is replenished and increases, making it more likely that a new epidemic will occur. This is the first study to show that the cyclical pattern of EV-A71 epidemics is associated with changes in EV-A71 seroprevalence.

## Introduction

Hand, foot and mouth disease (HFMD) is a common childhood disease, characterized by vesicles on the hands and feet, and ulcers in the mouth. Enterovirus A71 (EV-A71) is one of the main causative agents of HFMD apart from coxsackieviruses (CV) A6, A10, and A16 [1–3]. EV-A71, which belongs to the genus *Enterovirus* of the family *Picornaviridae*, is a small, non-enveloped, positive-stranded RNA virus. Substantial genetic diversity is observed in EV-A71. EV-A71 can be divided into three major genotypes, A, B and C, based on a cut-off nucleotide divergence value of 17–22% [4]. Genotypes B and C can be further subdivided into subgenotypes B0-B5 and C1-C5 respectively based on cut-off nucleotide divergence of 10–14% [4,5].

In addition to self-limiting HFMD or herpangina, EV-A71 is also rarely associated with more severe neurological diseases such as encephalitis, meningitis, acute flaccid paralysis and neurogenic pulmonary edema [6,7]. Since its first isolation in California in 1969, numerous epidemics of EV-A71 have been reported, mainly in Asia, including Singapore [8,9], Malaysia [10,11], Taiwan [12,13] and mainland China [14,15].

In Malaysia, HFMD epidemics due to EV-A71 were first documented in Sarawak, East Malaysia and Peninsular Malaysia in 1997, with over 2600 children affected and 48 deaths [10,11,16]. EV-A71 epidemics with fatalities recurred in Peninsular Malaysia in late 2000 [10] and in late 2005 [11], followed by an epidemic in Sarawak in early 2006, with 6 deaths reported [10,11]. Further epidemics of EV-A71 occurred in 2008/09 and 2012 [17–19]. In Sarawak, a clear 3-year recurrent cyclical pattern has been shown, with EV-A71 epidemics occurring in 1997, 2000, 2003 and 2006 [1,20]. A cyclical pattern of EV-A71 epidemics occurring every 3–4 years has also been described in Japan [21,22] and Singapore [23].

Cyclical epidemics may be due to various factors, including changes in pathogen antigenicity, variations in host population immunity, and environmental drivers [24]. Shifts in genotype often accompany new epidemics [18,21], but it is unclear whether these antigenic changes are the cause of recurrent epidemics. Most EV-A71 studies showed presence of cross-protective immunity against other genotypes following infection with a given genotype and high concordance in neutralization titers between the same genotypes [25–29]. Hence, changes in herd immunity are likely to be more important. As EV-A71 disease mainly affects young children below the age of 5, cyclical patterns of epidemics could be due to the accumulation of a new generation of susceptible children every few years, enabling sustained transmission [29]. However, direct evidence for this is scanty, as most EV-A71 seroprevalence studies are point prevalence studies.

In this study, we used 2,141 serum samples collected from children over an 18-year period encompassing 6 epidemics, and determined the association between seroprevalence rates and cyclical patterns of reported EV-A71 epidemics in Kuala Lumpur, Malaysia. We hypothesized that falls in EV-A71 seroprevalence rates were associated with new epidemics.

## Materials and Methods

### National HFMD data

Overall national incidence rates of notified HFMD from 2006 to 2012 were available from the Ministry of Health, Malaysia. However, as the statutory notification of HFMD came into enforcement only in October 2006, cases prior to this were underreported. The monthly numbers of HFMD cases for each of the 13 states and 2 federal territories were available only between 2008 and 2014. The case definition for reporting HFMD is a child with mouth/tongue ulcers and/or maculopapular rash/ vesicles on the palms and soles, with or without a history of fever. This data is syndromic, without laboratory confirmation of the viral agent. As diagnostic virology facilities are not widely accessible, there is scanty data on causative viral agents. Consequently, EV-A71 epidemic years, with limited laboratory confirmation, were obtained from published reports [10,11,16,19] and defined as 1997, 2000, 2003, 2006, 2008/2009, and 2012.

### Population and climatic data

Population data for Kuala Lumpur and Malaysia was generated based on the 2010 Population and Housing Census performed by the Department of Statistics, Malaysia [30]. Monthly climatic data for Kuala Lumpur consisting of temperature (°C), rainfall (mm), number of rain days and ultraviolet radiance (MJm^2^) were provided by the Malaysian Meteorological Department.

### Serum samples

Serum samples were randomly picked from archived residual sera collected for routine virology and bacteriology tests in the Diagnostic Virology Laboratory, University of Malaya Medical Center, in Kuala Lumpur, the capital of Malaysia. Samples from patients with suspected HFMD were excluded. A total of 1,769 sera from children aged between 1 to 12 years old, collected between 1995 and 2012, were tested for EV-A71 neutralizing antibodies. Between 52 and 200 samples were collected for each year, except for 2009, when only 30 samples from children were available. Samples were divided into 1–6 years (pre-school) and 7–12 years (primary school) age groups for most analyses. A further 372 serum samples from children <1 year were analyzed separately, as these may contain maternal antibodies.

### Ethics statement

The study was approved by the hospital’s Medical Ethics Committee (reference number 872.7) and the Medical Research and Ethics Committee of the Ministry of Health, Malaysia (reference number NMRR-12-1038-13816). Our institution does not require informed consent for retrospective studies of archived and anonymised samples.

### Neutralization assay

The selected serum samples were heat-inactivated at 56°C for 30 minutes. The neutralizing titer of each serum was determined by a microneutralization assay as described previously [8], with modifications. Two-fold serial dilution of each serum sample was performed from 1:8 to 1:32. An aliquot of 90 μl of each dilution was mixed with 90 μl of 1000 tissue culture infective dose (TCID_50_) of EV-A71 strain UH1/PM/97 (GenBank accession number AM396587) from subgenotype B4. The serum-virus mixture was then incubated at 37°C for 2 hours in 5% CO_2_. Each serum dilution was transferred into a 96-well plate in triplicate. A suspension of 100 μl containing 1 x 10^4^ rhabdomyosarcoma (RD, ATCC no. CRL-2061) cells was then added. Pooled positive sera of known titer were included in each assay as positive controls, using previously described criteria for reproducibility [8]. Wells containing diluted serum, virus alone, and uninfected RD cells were also included as controls. The plates were incubated at 37°C in 5% CO_2_ and examined for cytopathic effects (CPE) after 5 days. Neutralizing antibody titer was defined as the highest dilution that prevents the development of CPE in 50% of the inoculated cells. A sample was considered positive if the neutralizing titer was ≥1:8 [31,32]. Good cross-neutralization of serum against EV-A71 of different subgenotypes has been observed (S1 Table). Nevertheless, sera from children ≤3 years collected in 2013 were used to verify the concordance of neutralization titers between the UH1 strain and a clinical virus isolate from subgenotype B5 (GenBank accession number JN316092) isolated in 2006 (39 sera) and a clinical isolate from subgenotype C1 (GenBank accession number JN316071) cultured in 1997 (32 sera). These were the genotypes circulating in Malaysia between 1997–2012 [17]. High concordance in seropositive/seronegative status was obtained between UH1 and B5 virus (97%, 38/39 sera) and UH1 and C1 virus (81%, 26/32 sera); hence, this supports our use of the B4 virus alone for all the neutralization assays.

### Phylogenetic analysis and selective pressure analysis

We used phylogenetic analysis and selective pressure to investigate the role of genetic diversity in the cyclical patterns of EV-A71 epidemics. EV-A71 VP1 gene sequences of Malaysian isolates were retrieved from GenBank and aligned with Geneious R6 (Biomatters Ltd, New Zealand). A total of 275 VP1 sequences reported from Malaysia between 1997 and 2012 were available for analysis (S2 Table). The best substitution model was determined using jModelTest v0.1.1 [33] as the general time reversible model with rate variation among sites (GTR+G). Phylogenetic trees were constructed using the Bayesian Markov Chain Monte Carlo method in BEAST 1.7.4 [34], run for 30 million iterations with a 10% burn-in. All runs reached convergence with estimated sample sizes of >200. The clock model was uncorrelated lognormal relaxed and the tree prior was coalescent GMRF Bayesian Skyride, allowing the generation of a plot of relative genetic diversity, which reflects the change in effective population size over time [35]. The maximum clade credibility tree was viewed using FigTree 1.4 [36]. Selective pressure analysis was performed using codon-based maximum likelihood methods implemented in the Datamonkey web server [37]. Amino acids were only selected when positively identified by the two different codon-based maximum likelihood methods, which were single likelihood ancestor counting and fixed effects likelihood.

### Statistical analysis

We used cross-sectional time series analysis to determine the associations between epidemic periods and changes in seroprevalence. EV-A71 epidemic periods were categorized based on reported epidemic years obtained from limited laboratory confirmation and published reports [10,11,16,19]. We categorized time of our study into six distinct clusters: 1995–1997, 1998–2000, 2001–2003, 2004–2006, 2007–2009, and 2010–2012. A cluster of time consists of months before the epidemic and during the epidemic year. Epidemic periods were defined as the years 1997, 2000, 2003, 2006, 2008, 2009, and 2012. The seroprevalence rate was defined as the proportion of the samples tested which had a neutralization titer ≥1:8. The association of seroprevalence and epidemic months were modeled using generalized estimated equations population average models adjusted for confounders which are biologically plausible or have been previously described; these factors were age (1–6 years and 7–12 years), and climatic variables (monthly temperature, rainfall, rain days, and ultraviolet radiance). Interaction between seroprevalence, epidemic periods and age was also evaluated for possible heterogeneous effects or associations.

Geometric mean titers (GMT) were calculated by log-transforming the positive neutralization titers, using a value of 64 for titers >1:32. A two-sided type I error of 0.05 was used for statistical significance. Statistical analyses were performed using SPSS software version 22 (IBM SPSS Software, USA) and Stata version 12 (Stata Corp, College Station, Texas, USA), and graphs were drawn using GraphPad Prism 5 (GraphPad Software, USA).

## Results

### HFMD epidemics occur annually, mainly in young children

**Fig 1:**
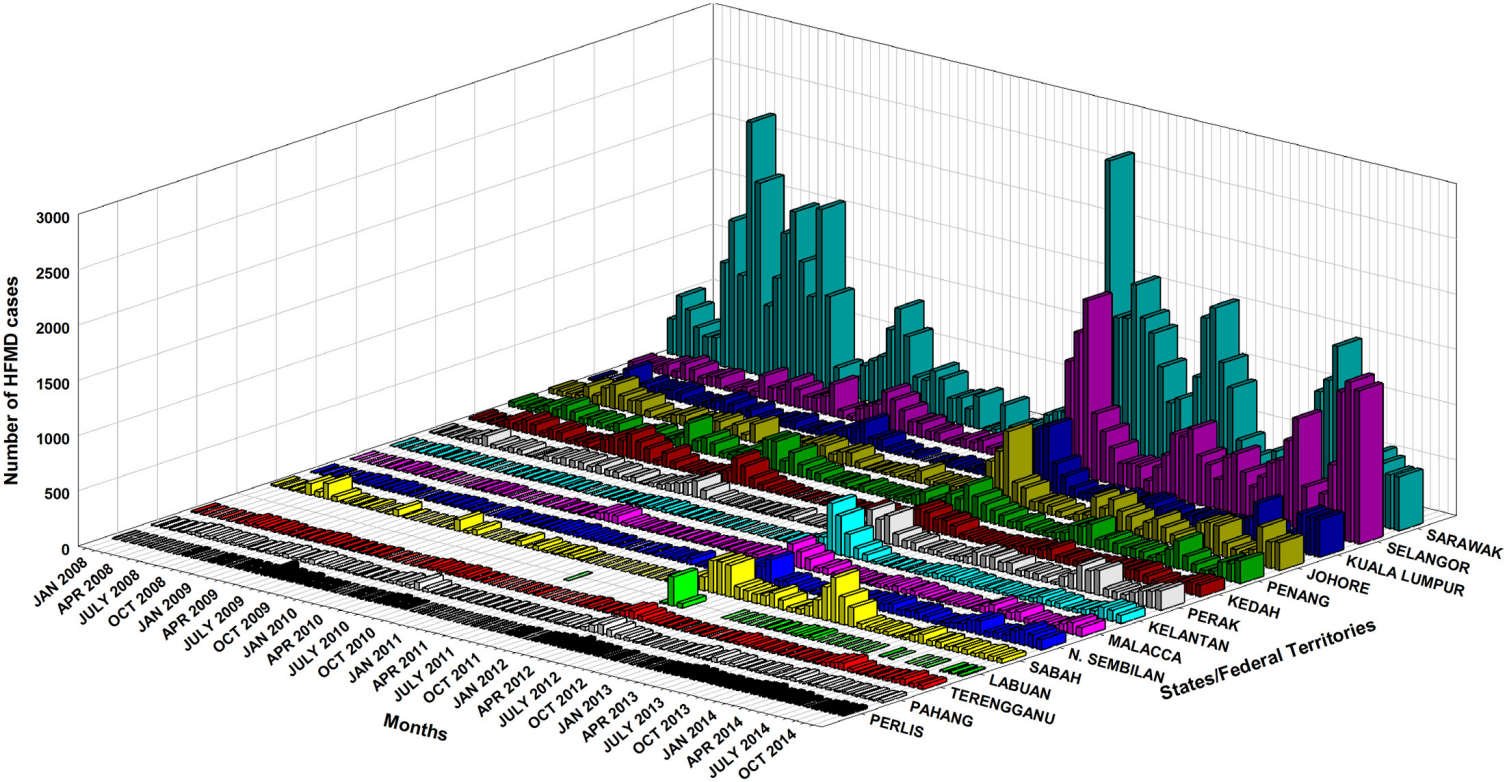
Monthly distribution of the number of HFMD cases in every state of Malaysia from 2008–2014. Data were obtained from Ministry of Health, Malaysia.

Malaysia consists of Peninsular Malaysia, where most of the country's 16 states and federal territories are located, and East Malaysia, which consists of Sabah, Sarawak and Labuan. The monthly notified HFMD cases in each state and federal territory were available from 2008–2014 (Fig 1). The total annual HFMD cases in 2008–2014 were 15,564, 17,154, 13,394, 7,002, 34,519, 23,331 and 31,322 respectively. In 5 of the 7 years, HFMD cases increased around March and peaked around May-June. Sarawak had the highest number of HFMD cases nationwide, while Selangor reported the highest number of cases among the states in Peninsular Malaysia.

Only national overall HFMD incidence rates were available from 2006. Details of causative viruses are generally not available, as most HFMD cases are clinically diagnosed and diagnostic virology facilities are not widely accessible. However, published reports of laboratory-confirmed EV-A71 epidemic years [2,3,11] were in accordance with cyclical reported HFMD activity from the available surveillance data, showing that EV-A71 epidemics occurred in Malaysia every 2–3 years, in 1997, 2000, 2003, 2006, 2008/9, and 2012.

**Fig 2:**
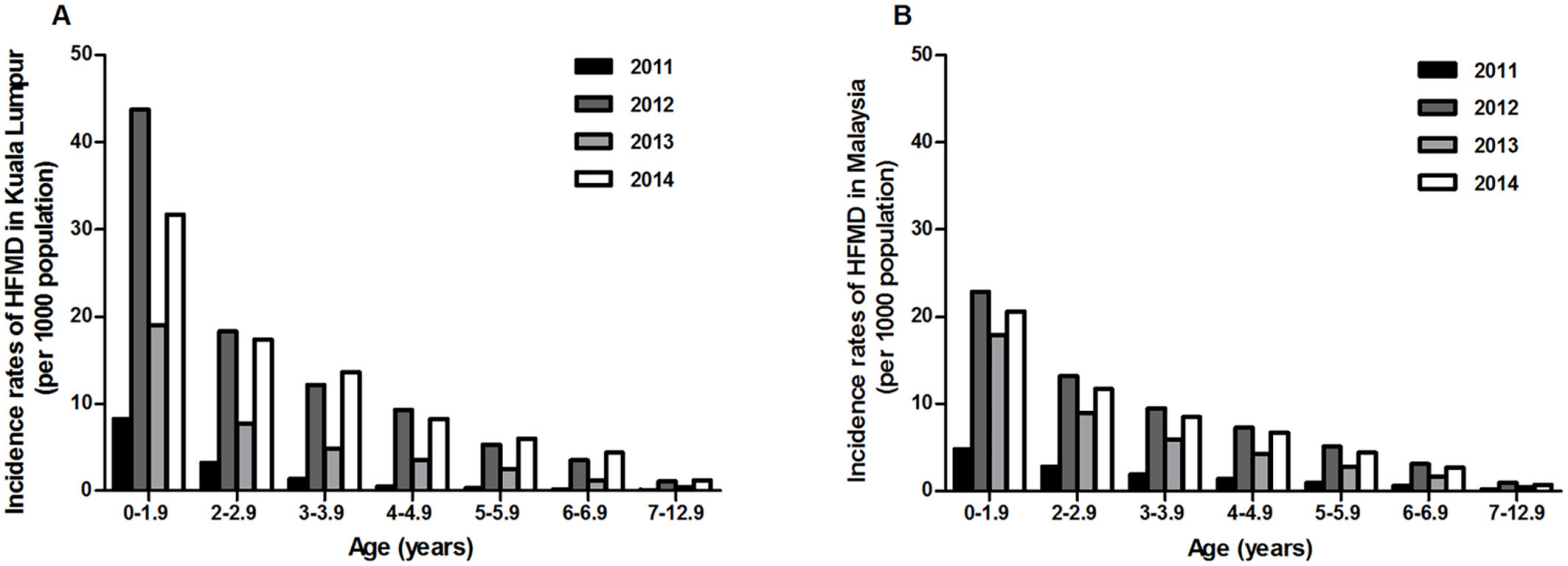
Age-specific incidence rates of HFMD cases in Kuala Lumpur and Malaysia. Incidence rates (per 1000 population) of HFMD cases in (A) Kuala Lumpur and (B) Malaysia, only available for 2011 to 2014, and obtained from Ministry of Health, Malaysia.

Age-specific incidence data was available only from 2011 to 2014 for the total HFMD cases nationwide and for the cases in Kuala Lumpur (Fig 2). The incidence rate of HFMD was the highest in those <2 years in both Kuala Lumpur (8.3, 43.7, 19.0 and 31.7 per 1000 population in 2011, 2012, 2013 and 2014, respectively) and Malaysia (4.8, 22.9, 17.9 and 20.6 per 1000 population in 2011, 2012, 2013 and 2014, respectively). The rates decreased with increasing age, with the 7–12 years age group having the lowest incidence rates; in Kuala Lumpur, rates were 0.07, 1.1, 0.4 and 1.2 per 1000 population in 2011, 2012, 2013 and 2014 respectively, and overall in Malaysia, rates were 0.2, 0.9, 0.4 and 0.7 per 1000 population in 2011, 2012, 2013 and 2014, respectively.

### EV-A71 seroprevalence and GMT decreases during non-epidemic periods

**Fig 3:**
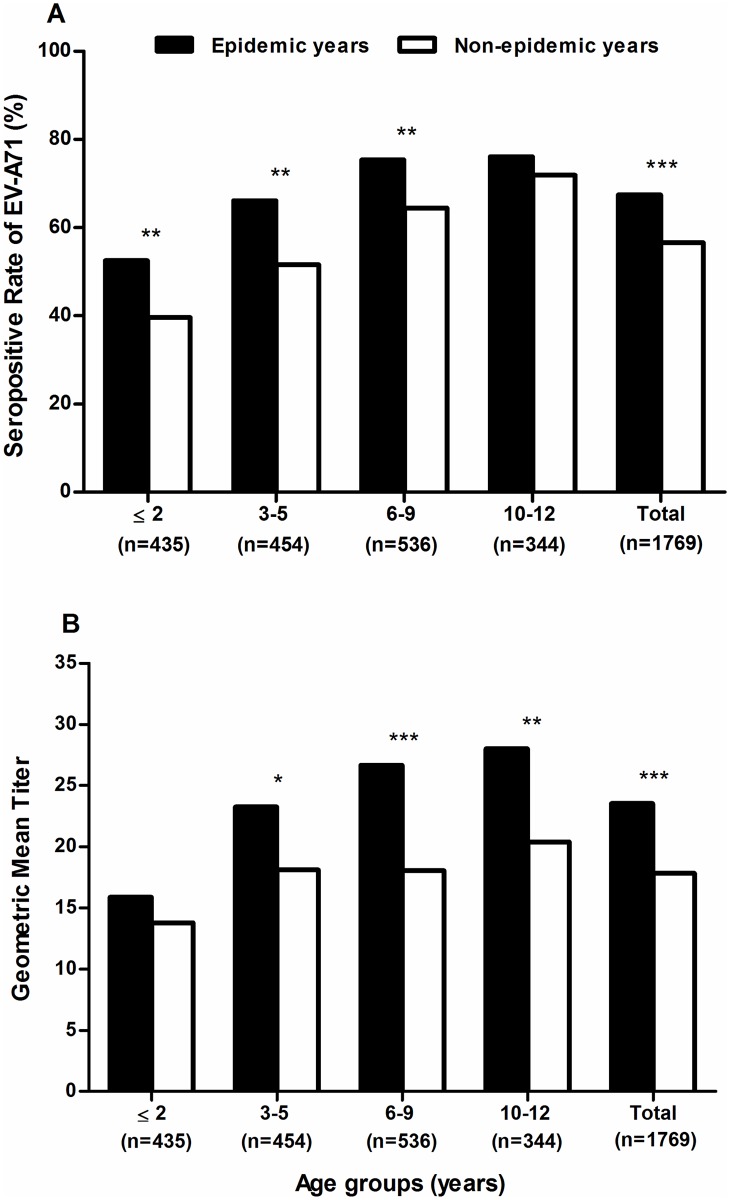
Age-specific seroprevalence and geometric mean titers of EV-A71 infection. (A) Seroprevalence rates and (B) geometric mean titers of EV-A71 infection by age, in epidemic and non-epidemic years (*P*<0.05*; *P*<0.01**; *P*<0.001***).

EV-A71 seroprevalence was higher in the primary school 7–12 years age group (71.6%, 95% CI 68.2–74.7%) compared to the preschool 1–6 years age group (52.8%, 95% CI 49.8–55.9%; *P*<0.001) overall, and in 16 out of the 18 years analyzed (significantly different in 3 years; S3 Table). The overall seroprevalence and GMT were significantly higher in epidemic years (seroprevalence 67.4%, 95% CI 63.8–70.9%; GMT 23.6, 95% CI 21.8–25.5) compared to non-epidemic years (seroprevalence 56.6%, 95% CI 53.6–59.5%; GMT 17.8, 95% CI 16.7–19.0; *P*<0.001) (Fig 3). During epidemic years, the seroprevalence of children aged 1–2 years (52.5%, 95% CI 44.8–60.0%), 3–5 years (66.1%, 95% CI 58.7–72.8%), and 6–9 years (75.4%, 95% CI 69.1–80.8%) were significantly higher compared to non-epidemic years (1–2 years old: 39.6%, 95% CI 33.9–45.5%; 3–5 years old: 51.6%, 95% CI 45.8–57.4%; and 6–9 years: 64.4%, 95% CI 59.1–69) (Fig 3A). GMT also rose significantly during epidemic years (3–5 years old: 23.3, 95% CI 19.8–27.3; 6–9 years: 26.7, 95% CI 23.4–30.4; and 10–12 years old: 28.0, 95% CI 23.5–33.4) compared to non-epidemic years (3–5 years old: 18.1, 95% CI 15.7–20.9; 6–9 years: 18.1, 95% CI 16.2–20.2; and 10–12 years old: 20.4, 95% CI 18.0–23.1) (Fig 3B).

**Fig 4:**
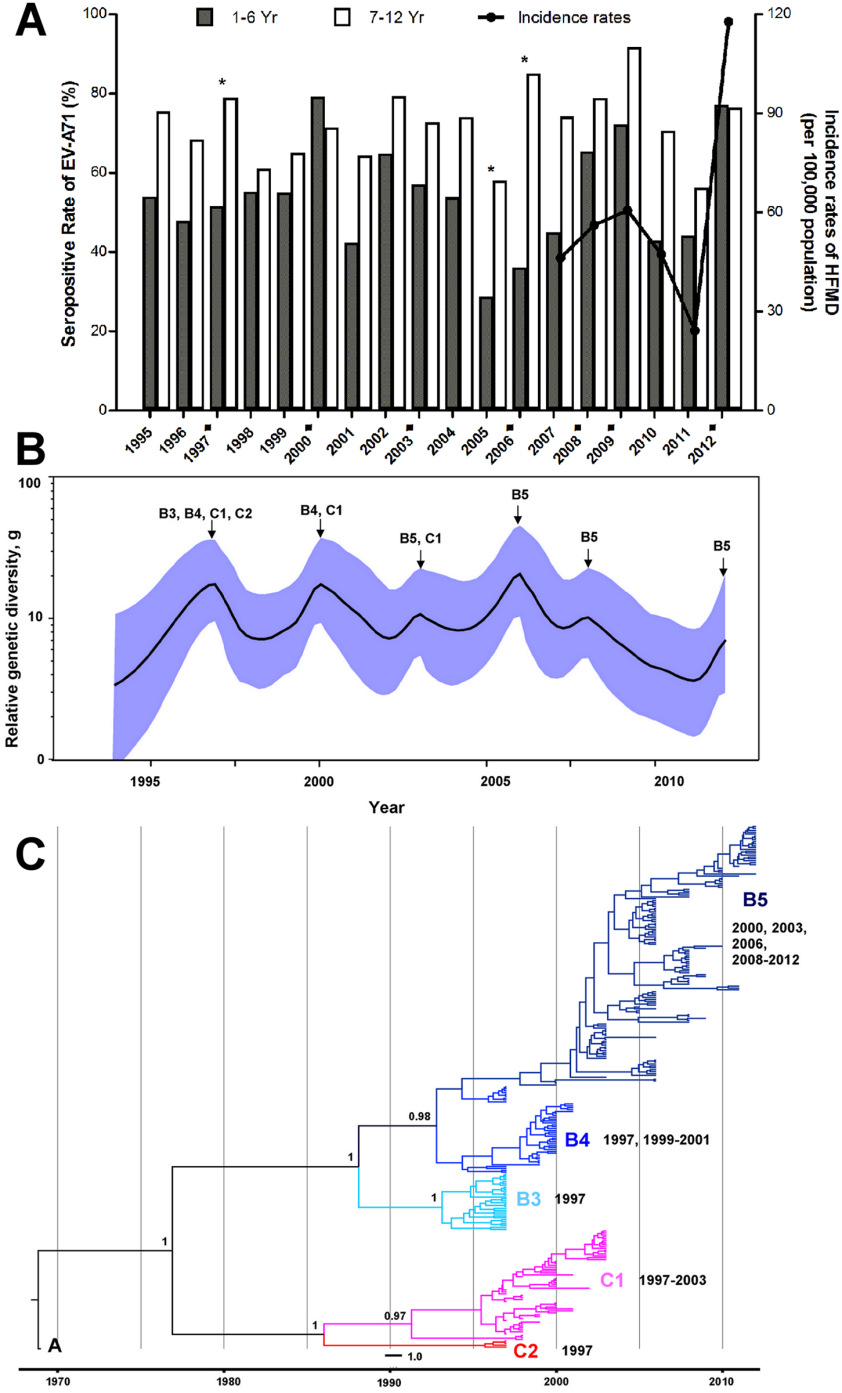
Age-dependent seroprevalence rates of EV-A71 infection in Kuala Lumpur, Malaysia from 1995–2012 and EV-A71 genetic diversity. (A) Seroprevalence rates of EV-A71 infection in 1–6 years (pre-school) and 7–12 years (primary school) age groups are shown. The asterisks indicate significant differences (*P*<0.05) in seroprevalence between the two age groups after Bonferroni correction for multiple comparisons across individual years. The black squares indicate reported EV-A71 epidemic years. The black line indicates the overall incidence rates of HFMD as reported by the Ministry of Health, Malaysia from 2007 (the statutory notification of HFMD was enforceable only from October 2006). (B) Bayesian Skyride plot estimating the genetic diversity of EV-A71 over time. (C) Maximum clade credibility tree based on VP1 sequences showing the EV-A71 subgenotypes present in Malaysia since the first epidemic in 1997. Posterior probability values are shown at the key nodes. EV-A71 BrCr from genotype A was used as the outgroup.

This is consistent with the observed general trend of EV-A71 seroprevalence spiking during reported EV-A71 epidemic years, and seroprevalence falling between epidemics (Fig 4A). These results showed that younger children aged 1–6 years old had lower seroprevalence in non-epidemic years, indicating greater susceptibility, which may explain the higher HFMD incidence in this age group (Fig 2). The higher seropositive rates and GMT levels seen during epidemic years are likely to reflect recent infection. HFMD incidence in older children aged 7–12 years is considerably lower; thus, the higher GMT levels seen during epidemics are more likely to represent re-exposure to EV-A71 or milder infection resulting in under-reporting. Taken together, both the incidence rates and the seroprevalence data suggested that HFMD caused by EV-A71 affects susceptible children aged 1–12 years, and most frequently affects younger children aged 1–6 years.

### EV-A71 phylogenetic analysis revealed emergence of different subgenotypes

Many subgenotypes were co-circulating during EV-A71 epidemics. Subgenotypes B3, B4, C1 and C2 were present during the 1997 epidemic, but only subgenotypes B4 and C1 continued to circulate till 2001 and 2003, respectively (Fig 4C). After 2003, subgenotype B5 became the sole genotype circulating in Malaysia. A Bayesian Skyride plot was used to estimate the evolutionary dynamics of EV-A71 in Malaysia over time (Fig 4B). Sharp, transient rises of genetic diversity were observed in the reported epidemic years 1997, 2000, 2003, 2006, 2008/2009, and 2012. The decline in the effective population seen after the 1997 epidemic may coincide with purifying selection against subgenotypes B3 and C2. The decline in the effective population observed after the 2000 and 2003 epidemics may indicate purifying selection against subgenotypes B4 and C1, respectively. After 2006, when only subgenotype B5 was circulating, interepidemic viral diversity showed overall decline punctuated by spikes during the epidemic years of 2008/2009 and 2012.

To further investigate the driving force of diversification in EV-A71, selective pressure on the Malaysian EV-A71 VP1 was examined. The mean dN/dS (ratio of nonsynonymous substitution rate to synonymous substitution rate) was 0.058, and evolution of EV-A71 was driven by strong purifying selection with over 62% of the analyzed codons under negative selection pressure. Two codons at positions 98 and 145 were under positive selective pressure, likely resulting in the emergence of new virus variants or lineage extinction. About 9.8% (27/275) of the sequences showed E98K, 3.6% (10/275) were E145Q, and 9.1% (25/275) were E145G and these could be observed in different genotypes across different EV-A71 epidemics (S2 Table).

These results showed that EV-A71 epidemics are characterized by peaks of increased genetic diversity, often with genotype changes. Evidence of strong negative selection and 2 codons with positive selection may explain the emergence of immune escape though the role in cyclical patterns of EV-A71 epidemics remains unclear.

### Changes in EV-A71 seroprevalence are associated with cyclical EV-A71 epidemics

Interaction between seroprevalence, age and epidemic periods was first evaluated in the model for possible heterogeneous effects in different strata of age groups. The association between a 10% increase in monthly seroprevalence and odds of an epidemic was 1.41 (95% CI 1.16–1.71) in those aged 1–6 years and 1.23 (95% CI 0.94–1.61) in those aged 7–12 years. The association between a 10% decrease between the preceding and current months and odds of an epidemic was 1.13 (95% CI 0.95–1.35) in those aged 1–6 years and 1.05 (95% CI 0.82–1.33) in those aged 7–12 years. The model incorporating the interaction terms of monthly seroprevalence with age groups and changes between seroprevalence of preceding and current months with age groups was tested, but showed non-significant interactions (*P* = 0.30). This suggests that age did not modify the association between the two measures of seroprevalence and epidemic period.

To further understand the relationship between recurrent EV-A71 epidemics and other factors such as seroprevalence, age and climatic variables, time series analysis was performed (Table 1). The monthly seroprevalence was positively associated with the odds of an epidemic period in the univariate analysis (OR for every 10% increase in seroprevalence, 1.40; 95% CI 1.18–1.65; *P*<0.001) and multivariate analysis after adjusting for plausible confounding factors such as age, temperature, rainfall, rain days, and ultraviolet radiance (adjusted OR for every 10% increase in seroprevalence, 1.45; 95% CI 1.24–1.69; *P*<0.001). This means that every 10% increase in monthly seroprevalence is associated with 45% higher odds of an epidemic, which is consistent with the observation that seroprevalence rates are higher during epidemics. We then examined whether relative changes in seroprevalence over time were associated with epidemics. Every 10% decrease in EV-A71 seroprevalence between preceding and current months was not significantly associated with epidemics in univariate analysis; but there was a significant association in multivariate analysis (aOR, 1.16; CI 1.01–1.35; *P*<0.034). This shows that every 10% fall in monthly seroprevalence compared to the preceding month is associated with 16% higher odds of an epidemic.

**Table 1:** Association between EV-A71 epidemics and seroprevalence in children from 1995–2012.

	Crude model		Adjusted model	
	OR (95% CI)	*P* value	OR (95% CI)	*P* value
10% increase in monthly seroprevalence	1.40 (1.18–1.65)	<0.001	1.45 (1.24–1.69)	<0.001
10% decrease in seroprevalence between preceding and current month	1.14 (0.99–1.32)	0.066	1.16 (1.01–1.34)	0.034
Age (7–12 years compared to 1–6 years)	0.55 (0.29–1.02)	0.060	0.65 (0.35–1.22)	0.182
Temperature (°C)	0.68 (0.43–1.06)	0.085	0.44 (0.29–0.68)	<0.001
Rainfall (mm)	1.00 (1.00–1.004)	0.17	1.00 (1.00–1.01)	0.137
Rain days	1.02 (0.95–1.09)	0.626	0.89 (0.79–0.99)	0.036
Ultraviolet radiance (MJm^2^)	1.01 (0.85–1.21)	0.874	1.07 (0.95–1.21)	0.276

## Discussion

In Asia, recurring epidemics of HFMD with associated severe neurological disease is a major public health concern. In Malaysia, HFMD became a statutorily notifiable disease only from October 2006, although national surveillance data does not include the causative viral agents. A notable exception is Sarawak, the worst affected state in Malaysia, which established sentinel and laboratory-based surveillance of HFMD in 1998, and clearly showed recurrent EV-A71 epidemics coinciding with large spikes in HFMD rates occurring at 2–3 year intervals [3,38]. We have found that national HFMD rates, which were not virus-specific, accorded with EV-A71 seroprevalence, spikes in genetic diversity of EV-A71, and published reports of laboratory-confirmed epidemic years. Together, this showed that EV-A71 epidemics also occurred in similar 3 year cycles in Malaysia. We found clear support for our hypothesis, showing that statistically significant decreases in population seroprevalence (as a proxy for immunity) are temporally associated with subsequent epidemics, after adjustment for age, temperature, rainfall, rain days, and ultraviolet radiance.

We identified seropositive children from as early as 1995 and 1996, suggesting that EV-A71 was already circulating before the first documented epidemic in 1997. The presence of seropositive young children in interepidemic years shows that ongoing transmission occurs between epidemics. This is supported by laboratory reports of EV-A71 isolated in low numbers during interepidemic years [3,12,17,39]. Based on the HFMD monthly distribution from 2008–2014, a seasonal pattern was observed, with incidence peaking between May to June. In USA, HFMD epidemics occur during summer and autumn months [40]. Taiwan has also showed higher incidence in the summer months [41] and in Guangzhou, incidence peaked in April/May and September/October [42]. The location-specific factors leading to seasonal epidemics have not been clearly defined, but could include climatic factors, such as the association with relative humidity and mean temperature in Taiwan [43], which may affect environmental survival of enteroviruses. In the present study, the overall likelihood of an epidemic was influenced by temperature and rain days, but not rainfall or ultraviolet radiance. The effects of these climatic factors on virus survival and spread will require further investigation. The relationships of HFMD with climatic variables remain to be explored in detail in Malaysia, particularly as individual states may have widely varying weather.

The highest age-specific incidence of HFMD is seen in children <2 years old (Fig 2). This is consistent with the significant differences in age-specific EV-A71 seroprevalence seen between non-epidemic and epidemic years in those <2 years old, particularly in the <6 month (from 47.7% to 64.0%, p = 0.016) and 6 months to 1 year age groups (from 35.9% to 64.3%, p = 0.0016). If an EV-A71 vaccine, such as the inactivated vaccine that has recently shown promise in phase 3 trials [44], were introduced into routine immunization programs, children would have to be vaccinated at least by the age of 6 months, and possibly earlier [45,46]. As most children in Malaysia and other Asian countries [9,31] are seropositive by 5 years, an effective vaccine could prevent EV-A71 HFMD, as well as the severe associated neurological complications that mainly affect the very young [47].

The well-recognized cyclical pattern of EV-A71 epidemics seen in some countries has been attributed to the time taken for accumulation of enough susceptible children in the population. In Tokyo, the overall EV-A71 seroprevalence dropped to its lowest point in 6 years during the months just preceding an epidemic in 1973, including an absence of antibodies in children <4 years old [48]. In Guangdong, China, seroprevalence gradually dropped from 2007 to 2009, before a large epidemic in 2010 [49]. In Taiwan, there was evidence of fewer EV71 seroconversions in 1994–1997, before the 1998 epidemic [47]. Our study is unusual as it charts seroprevalence over a long period of time, covering 18 years and 6 epidemics, and we showed that changes in population immunity in children appear to be the major driving force of the observed cyclical epidemics. Specifically, we demonstrated that falls in seroprevalence were clearly associated with higher odds of a subsequent epidemic. Seroprevalence in both 1–6 and 7–12 years age groups increased in epidemic years, suggesting that both groups are involved in disease burden and transmission. The higher HFMD rates seen in children aged 1–6 years is most likely due to their greater susceptibility (as shown by their lower seroprevalence rates in non-epidemic years), but it may also be due to under-reporting in older children, who often have milder disease [38,50].

Estimation of the basic reproduction ratio (R_0_), or the number of secondary cases arising from an infectious case, has been widely used to study the dynamics of transmission of infectious diseases such as SARS and influenza [51]. The R_0_ of EV-A71 has been estimated as 5.48, which is considered as moderately infectious [52]. The EV-A71 R_0_ was higher than the estimated CV-A16 R_0_ of 2.5, suggesting that EV-A71 is more transmissible. For such a transmissible virus, the epidemic size is mainly dependent on the size of the susceptible population [53]. Following a viral epidemic, most of the population at risk would become immune. It may then take 2–3 years for the susceptible population to be replenished by newborns, and to be large enough for the R_0_ to increase to >1, hence leading to a cyclical pattern of EV-A71 epidemics every 2–3 years. A similar study should be conducted to determine the R_0_ to further understand EV-A71 transmission dynamics in Malaysia.

The present study also showed that Malaysian epidemics are characterized by peaks of increased genetic diversity, often with genotype changes. While the increased diversity may simply reflect a larger number of infections, we cannot exclude that new variants with antigenic changes may escape population immunity and contribute to cyclical epidemics. Although found in less than a quarter of Malaysian EV-A71, the positive selection pressure sites found at positions 98 and 145 of the VP1 protein have been previously reported [5]. These mutations appeared at the terminal branches with changes from E98K, E145Q and E145G. Amino acid position 98 is part of the BC loop and position 155 is part of the DE loop, both of which are immunogenic loops of VP1 [17]. A recent study measured cross-reactive neutralizing antibody titers against viruses with mutations at residues 98, 145 and 164 [54]. Up to 4-fold neutralization reduction was seen in sera from children, adults and rabbits tested against an EV-A71 VP1-98K/145Q/164E mutant, and all neutralization titers were ≥ 1:16. However, viruses with all three mutations concomitantly have not yet been seen in nature. The significance of the antigenic variation will require more detailed longitudinal serological studies. If immune escape is not needed or plays only a minor role to produce the cyclical pattern of EV-A71 epidemics, a significant accumulation of susceptible children between epidemics will be enough to support large-scale transmission and another epidemic.

Overall, in other published studies, EV71-infected children have detectable neutralizing antibody titers against all the EV71 genotypes [27], and cross-protective immunity between genotypes is generally considered to be high [28,55]. Previous studies in humans, monkeys, rabbits and mice showed that neutralization antibody levels against different genotypes may vary, but overall human anti-serum generally does cross-neutralize strains of different genotypes (S1 Table). Lower neutralization titers may not reflect antigenic shift sufficient to lead to immune escape. To date, no cases of recurrent EV-A71 infection have been reported, suggesting the presence of life-long protective immunity against EV-A71. While enteroviruses clearly undergo antigenic evolution, complete immunological escape in EV-A71 seems to be rare, thus EV-A71 is generally considered to be a single serotype antigenically. Any possible clinical significance and contribution of reduced cross-protective immunity towards new epidemics will require further confirmation.

Our study's findings may be a useful basis for future efforts to forecast EV-A71 HFMD epidemics in Malaysia. Occurrence of EV-A71 epidemics may be predicted by seroprevalence rates in children and influenced by temperature and number of rain days. The changing population immunity, the effects of climate variables on the survival and spread of EV-A71 in the environment, the change in virus genetic diversity, and changing probability of transmission of EV-A71 due to changes in host behavior under certain climatic conditions may explain the seasonal cyclical patterns. Time-series analysis of real-time, high-quality surveillance and seroprevalence data may provide efficient detection and effective forecasting of EV-A71 epidemics. Future research may also focus on the potential influence of other HFMD enteroviruses in the cyclical pattern of EV-A71 epidemics.

The main limitation of this study is that we used a convenience sample of residual diagnostic sera from a single hospital. However, it would be difficult to otherwise obtain such an extensive collection of serum samples from healthy children over many years. When compared to a random cluster survey, convenience sampling has also been shown to give similar estimates of seroprevalence to 5 vaccine-preventable viral diseases [56]. The convenience sample used here is likely to be appropriate for this study.

In conclusion, falls in seroprevalence in children aged 1–12 years old are the major driving force of the cyclical pattern of EV-A71 epidemics seen in Malaysia over 18 years. Nevertheless, possible interplay between seroprevalence with climatic variables and virus antigenic variations is evident and warrant future study. The highest age-specific incidence of disease, as shown by surveillance figures and seroprevalence rates, occurred in children <2 years. Together with the seasonal and cyclical patterns observed, this study has provided important data which will impact vaccine planning, timing and target populations for vaccine programs.

## Supporting Information

S1 Table Summary of studies investigating the cross-reactivity neutralizing antibody responses to enterovirus A71 infection in humans and animals. (DOCX)

S2 Table Malaysian EV-A71 VP1 sequences from 1997–2012. (DOCX)

S3 Table Seroprevalence rates of EV-A71 neutralizing antibody in children, 1995–2012. (DOCX)

S1 Checklist STROBE checklist. (DOC)
