# Supplementary material for: Cyclical Patterns of Hand, Foot and Mouth Disease Caused by Enterovirus A71 in Malaysia
Source: PLoS Negl Trop Dis. 2016 Mar 24;10(3):e0004562. doi: 10.1371/journal.pntd.0004562 (PMC4806993; doi:10.1371/journal.pntd.0004562)
Supplement: S1 Table — (DOCX) [file pntd.0004562.s001.docx]

S1 Table: Summary of studies investigating the cross-reactivity of neutralizing antibody responses to enterovirus A71 (EV-A71) infection in humans and animals

A. Cross-reactivity in human sera:

| **Reference** | **EV-A71 strains/genotypes** | **Source of patient sera** | **Findings** |
| --- | --- | --- | --- |
| [1] | A  B3, B4, B5  C1, C2, C3, C4, C5 | 160 sera (40 individuals in each age group; 20 in vaccine group and 20 in placebo group) inoculated with inactivated EV-A71 vaccine (FY-23K-B strain of subgenotype C4) were collected at 0, 56 and 360 days post-vaccination for cross-neutralization assay. Individuals were aged 6-11 months, 12-23 months, 24-35 months and 36-71 months. | Cross-neutralizing reactivity against major EV-A71 strains was observed in the vaccine groups. All sera exhibited similar low neutralizing titers to the genotype C1 strain although these neutralizing titers were higher than the positive control titer of 1:8. |
| [2] | B4, B5  C2,C4a, C4b | 34/37 pediatric patients aged 0-18 years with enteroviral infections; 3 healthy children were enrolled as controls. | Infection with genotype B (B5) EV-A71 induced a strong genotype B EV-A71–specific but weaker genotype C (C4b)–specific antibody-secreting cell (ASC) response in children. Neutralization titers against genotype B viruses significantly correlated with genotype B EV-A71–specific ASC responses but not with genotype C EV-A71–specific ASC responses. |
| [3] | A, B5  C4a | 20 infected with B5 subgenotype and 10 infected with C4a subgenotype (≤3 years, 4-6 years and ≥7 years). | The 20 children infected with B5 viruses had significantly lower neutralizing antibody titers against genotype A virus and genotype C4a viruses than B5. |
| [4] | B4, B5  C2, C4, C5 | Sera from 119 infants and children (aged 6 months to 5 years) in two clinical trials of EV-A71 subgenotype C4 vaccine. | After two-dose vaccination, 49/53 (92.4%) participants in the initially seronegative group and 52/53 (98.1%) participants in the initially seropositive group showed < 4-fold differences in neutralizing titers against five EV-A71 strains, whereas corresponding values among sera from pediatric patients recovering from EV-A71-induced HFMD and subclinically infected participants were 8/8 (100%) and 41/43 (95.3%), respectively. The GMT of participants against five subgenotypes of EV-A71 increased significantly after vaccinations, irrespective of the baseline neutralizing antibody titer. |
| [5] | A  B1, B2, B3, B4, B5  C1, C2, C3, C4, C5 | Sera from children < 5 years, infected with genotypes:  C2 – 10 samples (1998)  B4 – 5 samples (2000)  C4 – 2 samples (2005)  B5 – 5 samples (2008)  C4 – 3 samples (2010) | Overall, all EV-A71-infected children had detectable neutralizing antibody titers against 11 EV-A71 genotypes. Homologous neutralizing antibodies titers were not always higher than heterologous neutralizing antibody titers. Children infected with genotypes C2, C4, B4 and B5 had lower GMTs (≥4-fold difference) against genotype A than other genotypes but no clear antigenic variations between genotype B and C were observed. |
| [6] | B1, B5  C2, C4A, C4B | Sera from 60 healthy adult vaccinees (aged between 20 and 60 years) were collected 0, 21 and 42 days post-vaccination. | Strong cross-neutralizing antibody responses in >85% of volunteers without pre-existing neutralizing antibodies against subgenotype B1, B5 and C4A were observed. Weaker cross-neutralizing antibody responses were found against C4B and C2 viruses. |
| [7] | A, B1, B4, B5  C2, C4, C5 | 5 groups of sera were collected:  Anti-C2 (3 samples)  Anti-B4 (4 samples)  Anti-C4 (4 samples)  Anti-C5 (1 sample)  Anti-B5 (2 samples) | Human antiserum showed various neutralization antibody titers against different viruses, indicating possible antigenic variations among viruses. The antigenic map showed that genotype B1 and B4 viruses clustered together while genotype C2 from 1998 was found in another antigenic cluster distinct from genotype B viruses. Genotype C4 viruses were more antigenically related to genotype C2. The reemergent genotype B5 viruses in 2003 and 2008 were antigenically different from the other genotype B and genotype C viruses but still showed neutralization against other genotypes. |
| [8] | A, B2, B4, B5  C1, C2, C4 | Sera from 83 residents in Yamagata aged 1 - > 60 years old were enrolled. | Residents previously infected with EV-A71 had neutralizing antibody against the different subgenotypes. The ranges of the neutralizing Ab titers against different subgenotype strains in the Yamagata residents differed by up to almost 4-fold. |

B. Cross-reactivity in animal sera (mice, rabbits, monkeys):

| **Reference** | **EV-A71 strains/genotypes** | **Sera** | **Findings** |
| --- | --- | --- | --- |
| [9] | C4 | Sera were collected from mice challenged with EV-A71. | An inactivated EV-A71 vaccine candidate offered complete protection from death induced by various circulating EV-A71 viruses to neonatal mice that were born to immunized female mice. The sera of the immunized dams and their pups showed high neutralization titers against multiple circulating EV-A71 viruses. |
| [10] | B3, B4, B5  C2, C4 | Rhesus macaques were immunized with EV-A71-B5 VLP vaccine at day 0 and 21. The macaques were challenged with EV-A71- B3 on day 42. Blood samples were collected and processed at different intervals post-challenge. | Sera from vaccinated animals neutralized EV-A71- B3 *in vitro*. The profile of neutralizing antibody titers was similar for all four subgenogroups (B4, B5, C2 and C4). All vaccinated animals produced neutralizing antibodies against EV-A71 from all subgenogroups tested. EV-A71-B5 VLPs elicited antibodies that could cross-neutralize multiple EV-A71 subgenotypes. EV-A71-B3 infection also induced neutralizing antibodies against other EV-A71 subgenotypes. |
| [11] | A  B, B2, B3, B4, B5  C1, C2, C3, C4, C5  C2-like | Sera from rabbits immunized with purified viruses collected a week after final boost. | The reference EV-A71 viruses induced high homotypic neutralization titers (1:256 to 1:4096). Genotype A virus consistently had >8-fold difference between homotypic and heterotypic neutralizing antibody titers but no clear pattern could be identified for genotype B and C viruses. Genotype B2 and B5 viruses were highly immunogenic and induced high homotypic and heterotypic neutralizing antibody titers against all genogroup B and C viruses except the C2-like virus isolated in 2008. |
| [12] | A  B, B2, B3, B4, B5  C1, C2, C3, C4, C5  (18 isolates) | 40 monoclonal antibodies were used to type the antigenic profiles of 18 isolates from different EV-A71 genotypes. | The antigenic profiles characterized by the monoclonal antibodies panel did not correlate to their genotypes by phylogenetic classification. Ten isolates of genotype C4a were classified into four distinct antigenic types, but the panel monoclonal antibodies still neutralised all virus genotypes. |
| [13] | B4, B5  C4 | Sera from mice and rabbit immunized with different EV-A71 immunogens (synthetic peptides, individual recombinant viral proteins, VLP, formalin-inactivated virions). | In mice, only VP1 elicited antibody responses with 1:128 neutralization titer. The formalin-inactivated EV-A71 elicited antibodies that cross-neutralized (1:640) different EV-A71 genotypes in mice. In rabbit, sera cross-neutralized strongly against different genotypes of EV-A71 (1:6400). |
| [14] | B3, B4,  C1, C2, C3, C4, C5 | Sera from mice immunized with formaldehyde-inactivated whole-virus vaccines derived from EV-A71 clinical isolates and mouse-adapted virus (MAV) were collected at day 14. | Antisera generated by immunization with the MAV vaccine not only neutralized genotype B3 EV-A71 strains (≥1:512), but also neutralized strains of genotypes B4 (≥1:512) and C1 to C5 (1:256, 1:64, 1:256, 1:32 and 1:64, respectively). |
| [15] | A  B0, B1, B1*, B2  C2 | Sera from rabbits hyperimmunized with EV-A71 isolates of subgenotype B2 and C1. | Subgenotype B2-specific rabbit antiserum showed cross-neutralization of B0, B1 and B2, but very low neutralization against subgenogroup C1 or C2 viruses, probably explaining the global shift to genogroup C in 1987 following a B2 epidemic. Anti-C1 rabbit serum neutralized both genogroup B and C viruses. |
| [16] | A  B1, B4  C2, C4 | Sera from monkeys inoculated with EV-A71, followed by lethal challenge with the parental virulent strain EV-A71 (BrCr-TR) of genotype A. | The immunized monkey sera showed a broad spectrum of neutralizing activity against different genotypes of EV-A71, including genotypes A, B1, B4, C2, and C4. The sera showed the highest homotypic neutralization activity (genotype A) and the lowest neutralization activity against genotype C2. The order of decreasing neutralization activity of sera was: A>B1>C4>B4>C2. |

**References and bibliographic details**

1. Liu L, Mo Z, Ling Z, Zhang Y, Li R, Ong KC, et al. Immunity and clinical efficacy of an inactivated enterovirus 71 vaccine in healthy Chinese children: a report of further observations. BMC Med. 2015; 13: 226.
2. Huang KY, Lin JJ, Chiu CH, Yang S, Tsao KC, Huang YC, et al. A potent virus-specific antibody-secreting cell response to acute enterovirus 71 infection in children. J Infect Dis. 2015; 212: 808-817.
3. Luo ST, Chiang PS, Chung WY, Chia MY, Tsao KC, Wang YH, et al. Reemergence of enterovirus 71 epidemic in Northern Taiwan, 2012. PLoS ONE. 2015; 10: e0116322.
4. Mao Q, Cheng T, Zhu F, Li J, Wang Y, Li Y, et al. The cross-neutralizing activity of enterovirus 71 sugenotype C4 vaccines in healthy infants and children. PLoS ONE. 2013; 8: e79599.
5. Huang ML, Chiang PS, Chia MY, Luo ST, Chang LY, Lin TY, et al. Cross-reactive neutralizing antibody responses to enterovirus 71 infections in young children: implications for vaccine development. PLoS Neg Trop Dis. 2013; 7: e2067.
6. Chou AH, Liu CC, Chang JY, Jiang R, Hsieh YC, Tsao A, et al. Formalin-inactivated EV71 vaccine candidate induced cross-neutralizing antibody against subgenotypes B1, B4, B5 and C4A in adult volunteers. PLoS ONE. 2013; 8: e79783.
7. Huang SW, Hsu YW, Smith DJ, Kiang D, Tsai HP, Lin KH, et al. Reemergence of enterovirus 71 in 2008 in Taiwan: Dynamics of genetic and antigenic evolution from 1998 to 2008. J Clin Microbiol. 2009; 47: 3653-3662.
8. Mizuta K, Aoki Y, Suto A, Ootani K, Katsushima N, Itagaki T, et al. Cross-antigenicity among EV71 strains from different genogroups isolated in Yamagata, Japan, between 1990 and 2007. Vaccine. 2009; 27: 3153-3158.
9. Chang J, Li J, Liu X, Liu G, Yang J, Wei W, et al. Broad protection with an inactivated vaccine against primary-isolated lethal enterovirus 71 infection in newborn mice. BMC Microbiol. 2015; 15: 139.
10. Lim PY, Hickey AC, Jamiluddin MF, Hamid S, Kramer J, Santos R, et al. Immunogenicity and performance of an enterovirus 71 virus-like-particle vaccine in nonhuman primates. Vaccine. 2015; 44: 6017-6024.
11. Chia MY, Chung WY, Chiang PS, Chien YS, Ho MS, Lee MS, et al. Monitoring antigenic variations of enterovirus 71: implications for virus surveillance and vaccine development. PLoS Negl Trop Dis. 2014; 8: e3044.
12. Chen Y, Li C, He D, Cheng T, Ge S, Shih JW, et al. Antigenic analysis of divergent genotypes human enterovirus 71 viruses by a panel of neutralizing monoclonal antibodies: Current genotyping of EV71 does not reflect their antigenicity. Vaccine 2013; 31: 425-430.
13. Chou AH, Liu CC, Chang JY, Lien SP, Guo MS, Tasi HP, et al. Immunological evaluation and comparison of different vaccine candidates. Clin Dev Immunol. 2012; 2012: 831282.
14. Ong KC, Devi S, Cardosa MJ, Wong KT. Formaldehyde-inactivated whole-virus vaccine protects a murine model of enterovirus 71 encephalomyelitis against disease. J Virol. 2010; 84: 661-665.
15. van der Sanden S, van der Avoort H, Lemey P, Uslu G, Koopmans M. Evolutionary trajectory of the VP1 gene of human enterovirus 71 genogroup B and C viruses. J Gen Virol. 2010; 91: 1949-1958.
16. Arita M, Nagata N, Iwata N, Ami Y, Suzaki Y, Mizuta K, et al. An attenuated strain of enterovirus 71 belonging to genotype A showed a broad spectrum of antigenicity with attenuated neurovirulence in cynomolgus monkeys. J Virol. 2007; 81: 9386-9395.
