# Supplementary material for: Cyclical Patterns of Hand, Foot and Mouth Disease Caused by Enterovirus A71 in Malaysia
Source: PLoS Negl Trop Dis. 2016 Mar 24;10(3):e0004562. doi: 10.1371/journal.pntd.0004562 (PMC4806993; doi:10.1371/journal.pntd.0004562)
Supplement: S2 Table — (DOCX) [file pntd.0004562.s002.docx]

S2 Table: Malaysian EV-A71 VP1 sequences from 1997-2012

| Accession  number | Name | Year of  isolation | Subgenotype | Positive selective pressure sites | References |
| --- | --- | --- | --- | --- | --- |
| AF190565 | 1109_MAA_1997 | 1997 | C1 | E98K | [1] |
| AF190567 | 1112_MAA_1997 | 1997 | C1 | - | [1] |
| AY207612 | 03784_MAA_1997 | 1997 | C1 | - | [2] |
| JN316071 | PM_14283_1997 | 1997 | C1 | - | [3] |
| AF190568 | 1113_MAA_1997 | 1997 | C1 | - | [1] |
| AY207638 | 0283_MAA_1997 | 1997 | C1 | E98K | [2] |
| AY207615 | 03750_MAA_1997 | 1997 | C2 | - | [2] |
| AM396584 | Sha52_1997 | 1997 | C2 | - | [4] |
| AM396585 | Sha71_1997 | 1997 | C2 | - | [4] |
| AY207611 | 03907_MAA_1997 | 1997 | C2 | - | [2] |
| AY207649 | 0091_MAA_1997 | 1997 | B4 | E98K | [2] |
| AF190560 | 1103_MAA_1997 | 1997 | B4 | E98K | [1] |
| AF190559 | 1102_MAA_1997 | 1997 | B4 | E98K | [1] |
| JN316108 | PM_13091_1997 | 1997 | B4 | E98K | [3] |
| AY207646 | 0414_MAA_1997 | 1997 | B4 | E98K | [2] |
| AM396587 | UH1_1997 | 1997 | B4 | E98K | [4] |
| AY207647 | 0128_MAA_1997 | 1997 | B4 | - | [2] |
| AY207640 | 0175_MAA_1997 | 1997 | B4 | E98K | [2] |
| AF190566 | 1110_MAA_1997 | 1997 | B4 | - | [1] |
| AY207637 | 0343_MAA_1997 | 1997 | B4 | E98K | [2] |
| AY207613 | 03300_MAA_1997 | 1997 | B4 | - | [2] |
| AY207643 | 0898_MAA_1997 | 1997 | B4 | E145Q | [2] |
| AJ586873 | Sha89_1997 | 1997 | B4 | - | [4] |
| JN316109 | PM_13899_1997 | 1997 | B3 | E145G | [3] |
| AF190561 | 1105_MAA_1997 | 1997 | B3 | E145G | [1] |
| AY207644 | 0897_MAA_1997 | 1997 | B3 | E145G | [2] |
| JN316110 | PM_13473_1997 | 1997 | B3 | - | [3] |
| AF190564 | 1108_MAA_1997 | 1997 | B3 | - | [1] |
| AY207616 | 0473_MAA_1997 | 1997 | B3 | - | [2] |
| AY207642 | 0899_MAA_1997 | 1997 | B3 | E145G | [2] |
| AY207648 | 0903_MAA_1997 | 1997 | B3 | E145G | [2] |
| AY207641 | 0036_MAA_1997 | 1997 | B3 | E145G | [2] |
| AY207645 | 0884_MAA_1997 | 1997 | B3 | E145G | [2] |
| AF190563 | 1107_MAA_1997 | 1997 | B3 | - | [1] |
| AF190562 | 1106_MAA_1997 | 1997 | B3 | - | [1] |
| AY207639 | 0245_MAA_1997 | 1997 | B3 | E98K | [2] |
| JN316111 | PM_14716_1997 | 1997 | B3 | E98K | [3] |
| AY207636 | 04716_MAA_1997 | 1997 | B3 | E145G | [2] |
| AF190570 | 2334_MAA_1997 | 1997 | B3 | - | [1] |
| AF190569 | 2294_MAA_1997 | 1997 | B3 | - | [1] |
| AY207614 | 0870_MAA_1997 | 1997 | B3 | - | [2] |
| AF376072 | MY104_9_SAR_1997 | 1997 | B3 | - | [5] |
| AB469182 | SK_EV006_1997 | 1997 | B3 | E145G | [6] |
| DQ341367 | MY821_3_SAR_1997 | 1997 | B3 | E145G | [7] |
| AF376076 | MY755_3_SAR_1997 | 1997 | B3 | E145G | [5] |
| AM396588 | Sha63_1997 | 1997 | B3 | - | [4] |
| AF190571 | 7202_MAA_1997 | 1997 | B3 | - | [1] |
| AF376078 | MY860_3_SAR_1997 | 1997 | B3 | - | [5] |
| DQ341368 | MY104_9_SAR_1997 | 1997 | B3 | E145Q | [7] |
| AF376074 | MY21_2_SAR_1997 | 1997 | B3 | E145G | [5] |
| AF376073 | MY16_1_SAR_1997 | 1997 | B3 | E145G | [5] |
| AM396586 | Sha66_1997 | 1997 | B3 | E98K | [4] |
| AF376075 | MY6_2_SAR_1997 | 1997 | B3 | E145G | [5] |
| AF190576 | 1118_MAA_1998 | 1998 | C1 | - | [1] |
| JN316067 | PM_17557_1998 | 1998 | C1 | - | [3] |
| AY207631 | 0557_MAA_1998 | 1998 | C1 | E98K | [2] |
| AY207630 | 0808_MAA_1998 | 1998 | C1 | E98K | [2] |
| JN316068 | PM_17838_1998 | 1998 | C1 | - | [3] |
| AF190574 | 1116_MAA_1998 | 1998 | C1 | - | [1] |
| AF190573 | 1115_MAA_1998 | 1998 | C1 | - | [1] |
| AF190575 | 1117_MAA_1998 | 1998 | C1 | - | [1] |
| AF190572 | 1114_MAA_1998 | 1998 | C1 | - | [1] |
| JN316069 | PM_17808_1998 | 1998 | C1 | - | [3] |
| AF376080 | S10862_SAR_1998 | 1998 | C1 | - | [5] |
| AF376079 | S10822_SAR_1998 | 1998 | C1 | - | [5] |
| AF376081 | S11051_SAR_1998 | 1998 | C1 | E98K | [5] |
| AY207629 | 0838_MAA_1999 | 1999 | C1 | E98K | [2] |
| JN316070 | PM_10749_1999 | 1999 | C1 | - | [3] |
| AY207653 | 0749_MAA_1999 | 1999 | C1 | E98K | [2] |
| JN316105 | PM_12627_1999 | 1999 | B4 | - | [3] |
| JN316104 | PM_12615_1999 | 1999 | B4 | - | [3] |
| JN316106 | PM_12919_1999 | 1999 | B4 | - | [3] |
| AY207650 | 0919_MAA_1999 | 1999 | B4 | - | [2] |
| AY207651 | 0627_MAA_1999 | 1999 | B4 | - | [2] |
| AY207652 | 0615_MAA_1999 | 1999 | B4 | E98K | [2] |
| AY207626 | 0389_MAA_2000 | 2000 | C1 | - | [2] |
| JN316076 | PM_17113_2000 | 2000 | C1 | - | [3] |
| AY207618 | 0807_MAA_2000 | 2000 | C1 | - | [2] |
| AY207625 | 0113_MAA_2000 | 2000 | C1 | - | [2] |
| JN316077 | PM_17204_2000 | 2000 | C1 | - | [3] |
| JN316073 | PM_15948_2000 | 2000 | C1 | - | [3] |
| AY207622 | 0948_MAA_2000 | 2000 | C1 | - | [2] |
| AY207620 | 0915_MAA_2000 | 2000 | C1 | - | [2] |
| AY207619 | 0836_MAA_2000 | 2000 | C1 | - | [2] |
| AF376087 | S40221_SAR_2000 | 2000 | C1 | - | [5] |
| AY207621 | 0937_MAA_2000 | 2000 | C1 | - | [2] |
| AY207632 | 0832_MAA_2000 | 2000 | C1 | - | [2] |
| JN316079 | PM_15774_2000 | 2000 | C1 | - | [3] |
| AY207634 | 0774_MAA_2000 | 2000 | C1 | - | [2] |
| JN316080 | PM_17181_2000 | 2000 | C1 | - | [3] |
| AY207635 | 05716_MAA_2000 | 2000 | C1 | - | [2] |
| JN316095 | PM_17177_2000 | 2000 | B5 | - | [3] |
| AY207633 | 0815_MAA_2000 | 2000 | B5 | E98K | [2] |
| JN316097 | PM_17467_2000 | 2000 | B4 | - | [3] |
| AY207628 | 0467_MAA_2000 | 2000 | B4 | - | [2] |
| AY207624 | 0066_MAA_2000 | 2000 | B4 | - | [2] |
| JN316099 | PM_17164_2000 | 2000 | B4 | - | [3] |
| AY207617 | 0778_MAA_2000 | 2000 | B4 | - | [2] |
| JN316100 | PM_17431_2000 | 2000 | B4 | - | [3] |
| AY207627 | 0431_MAA_2000 | 2000 | B4 | - | [2] |
| AY207623 | 0042_MAA_2000 | 2000 | B4 | - | [2] |
| AF376084 | S21082_SAR_2000 | 2000 | B4 | - | [5] |
| AF376067 | CN04104_SAR_2000 | 2000 | B4 | E145Q | [5] |
| AF376069 | SB0635_SAR_2000 | 2000 | B4 | - | [5] |
| JN316107 | PM_16042_2000 | 2000 | B4 | - | [3] |
| AF376083 | S12502_SAR_2000 | 2000 | B4 | - | [5] |
| AF376066 | SB2864_SAR_2000 | 2000 | B4 | - | [5] |
| AF376082 | S12172_SAR_2000 | 2000 | B4 | - | [5] |
| AF376085 | S2861_SAR_2000 | 2000 | B4 | - | [5] |
| AF376071 | CN9502_SAR_2000 | 2000 | B4 | - | [5] |
| AF376086 | S40201_SAR_2000 | 2000 | B4 | E145Q | [5] |
| AF376065 | SB1647_SAR_2000 | 2000 | B4 | - | [5] |
| AF376068 | CN062334_SAR_2000 | 2000 | B4 | - | [5] |
| AF376064 | SB1191_SAR_2000 | 2000 | B4 | - | [5] |
| AF376070 | CN0942_SAR_2000 | 2000 | B4 | E145G | [5] |
| JN316072 | PM_19552_2001 | 2001 | C1 | - | [3] |
| JN316081 | PM_19229_2001 | 2001 | C1 | - | [3] |
| DQ341360 | J115_MAL_2001 | 2001 | C1 | - | [7] |
| JN316098 | PM_20822_2001 | 2001 | B4 | E98K | [3] |
| JN316101 | PM_20680_2001 | 2001 | B4 | - | [3] |
| JN316102 | PM_20045_2001 | 2001 | B4 | - | [3] |
| JN316103 | PM_20756_2001 | 2001 | B4 | - | [3] |
| DQ341365 | PP37_MAL_2001 | 2001 | B4 | E145Q | [7] |
| AY189154 | S18191_SAR_2002 | 2002 | C1 | - | [5] |
| AY258316 | CN30552_SAR_2003 | 2003 | C1 | - | [7] |
| AY258300 | SB9522_SAR_2003 | 2003 | C1 | - | [8] |
| AY258296 | SB9582_SAR_2003 | 2003 | C1 | - | [7] |
| AY258317 | CN30014_SAR_2003 | 2003 | C1 | - | [7] |
| AY258298 | SB9564_SAR_2003 | 2003 | C1 | - | [7] |
| AY258297 | SB9579_SAR_2003 | 2003 | C1 | - | [7] |
| AY258302 | SB9465_SAR_2003 | 2003 | C1 | - | [7] |
| AY258299 | SB9533_SAR_2003 | 2003 | C1 | - | [7] |
| AY258301 | SB9508_SAR_2003 | 2003 | C1 | - | [7] |
| AY258315 | S19691_SAR_2003 | 2003 | C1 | - | [7] |
| AY258312 | S19761_SAR_2003 | 2003 | C1 | - | [7] |
| AY258295 | SB9604_SAR_2003 | 2003 | C1 | - | [7] |
| AY258314 | S19731_SAR_2003 | 2003 | C1 | - | [7] |
| AY258294 | SB9869_SAR_2003 | 2003 | C1 | - | [7] |
| JN316074 | PM_25405_2003 | 2003 | C1 | - | [3] |
| JN316075 | PM_24886_2003 | 2003 | C1 | - | [3] |
| DQ341363 | S19841_SAR_2003 | 2003 | B5 | - | [7] |
| AY258308 | S23141_SAR_2003 | 2003 | B5 | - | [7] |
| AY258309 | S19871_SAR_2003 | 2003 | B5 | - | [7] |
| AY258307 | S110031_SAR_2003 | 2003 | B5 | - | [7] |
| AY905550 | SB10712_SAR_2003 | 2003 | B5 | - | [8] |
| AY258313 | S19741_SAR_2003 | 2003 | B5 | - | [7] |
| AY258306 | S110101_SAR_2003 | 2003 | B5 | - | [7] |
| AY258303 | S110261_SAR_2003 | 2003 | B5 | - | [7] |
| AY258304 | S110241_SAR_2003 | 2003 | B5 | - | [7] |
| AY258305 | S110121_SAR_2003 | 2003 | B5 | - | [7] |
| AY905546 | SB12282_SAR_2003 | 2003 | B5 | - | [8] |
| AY905545 | SB12869_SAR_2003 | 2003 | B5 | - | [8] |
| AY905549 | SB11977_SAR_2003 | 2003 | B5 | - | [8] |
| AY905547 | SB12278_SAR_2003 | 2003 | B5 | - | [8] |
| AY258311 | S19791_SAR_2003 | 2003 | B5 | - | [7] |
| AY258310 | S19841_SAR_2003 | 2003 | B5 | E145G | [7] |
| DQ341362 | SB12736_SAR_2003 | 2003 | B5 | - | [7] |
| AY905548 | SB12007_SAR_2003 | 2003 | B5 | - | [8] |
| JN316083 | PM_26165_2003 | 2003 | B5 | - | [3] |
| JN316084 | PM_33034_2005 | 2005 | B5 | - | [3] |
| JN316085 | PM_32286_2005 | 2005 | B5 | - | [3] |
| JN316086 | PM_32308_2005 | 2005 | B5 | - | [3] |
| JN316082 | PM_34589_2006 | 2006 | B5 | E98K | [3] |
| FM201324 | EV71_MY1764589_2006 | 2006 | B5 | - | [9] |
| HQ676263 | MY46_Sw_A_2006 | 2006 | B5 | - | [10] |
| HQ676262 | MY45_Sw_A_2006 | 2006 | B5 | - | [10] |
| HQ676235 | MY17_Sw_A_2006 | 2006 | B5 | E145G | [10] |
| HQ676254 | MY37_Sw_A_2006 | 2006 | B5 | E145G | [10] |
| HQ676267 | MY98_Sw_A_2006 | 2006 | B5 | - | [10] |
| FM201322 | EV71_MY1764283_2006 | 2006 | B5 | - | [9] |
| FM201321 | EV71_MY1764281_2006 | 2006 | B5 | - | [9] |
| HQ676252 | MY34_Sw_A_2006 | 2006 | B5 | E145Q | [10] |
| FM201327 | EV71_MY1765058_2006 | 2006 | B5 | - | [9] |
| FM201326 | EV71_MY1760517_2006 | 2006 | B5 | - | [9] |
| JN316090 | PM_1687413_2006 | 2006 | B5 | E98K | [3] |
| JN316091 | PM_1657636_2006 | 2006 | B5 | E98K | [3] |
| JN316093 | PM_35017_2006 | 2006 | B5 | - | [3] |
| JN316092 | PM_1657640_2006 | 2006 | B5 | - | [3] |
| HQ676255 | MY38_Sw_A_2006 | 2006 | B5 | - | [10] |
| HQ676245 | MY27_Sw_A_2006 | 2006 | B5 | - | [10] |
| HQ676260 | MY43_Sw_A_2006 | 2006 | B5 | - | [10] |
| HQ676240 | MY22_Sw_A_2006 | 2006 | B5 | - | [10] |
| HQ676259 | MY42_Sw_A_2006 | 2006 | B5 | - | [10] |
| HQ676247 | MY29_Sw_A_2006 | 2006 | B5 | - | [10] |
| HQ676241 | MY23_Sw_A_2006 | 2006 | B5 | - | [10] |
| HQ676261_ | MY44_Sw_A_2006 | 2006 | B5 | - | [10] |
| HQ676246 | MY28_Sw_A_2006 | 2006 | B5 | - | [10] |
| JN316094 | PM_1673313_2006 | 2006 | B5 | - | [3] |
| HQ676258 | MY41_Sw_A_2006 | 2006 | B5 | E145Q | [10] |
| HQ676248 | MY30_Sw_A_2006 | 2006 | B5 | E145Q | [10] |
| HQ676242 | MY24_Sw_A_2006 | 2006 | B5 | E145Q | [10] |
| HQ676257 | MY40_Sw_A_2006 | 2006 | B5 | - | [10] |
| HQ676256 | MY39_Sw_A_2006 | 2006 | B5 | - | [10] |
| HQ676244 | MY26_Sw_A_2006 | 2006 | B5 | - | [10] |
| HQ676239 | MY21_Sw_A_2006 | 2006 | B5 | - | [10] |
| HQ676249 | MY31_Sw_A_2006 | 2006 | B5 | - | [10] |
| HQ676236 | MY18_Sw_A_2006 | 2006 | B5 | E145G | [10] |
| HQ676234 | MY16_Sw_A_2006 | 2006 | B5 | E145G | [10] |
| HQ676250 | MY32_Sw_A_2006 | 2006 | B5 | - | [10] |
| HQ676243 | MY25_Sw_A_2006 | 2006 | B5 | - | [10] |
| HQ676253 | MY35_Sw_A_2006 | 2006 | B5 | - | [10] |
| HQ676266 | MY97_Sw_A_2006 | 2006 | B5 | - | [10] |
| HQ676251 | MY33_Sw_A_2006 | 2006 | B5 | - | [10] |
| HQ676237 | MY19_Sw_A_2006 | 2006 | B5 | - | [10] |
| FM201325 | EV71_MY_2006 | 2006 | B5 | - | [9] |
| JN316088 | PM_34242_2006 | 2006 | B5 | - | [3] |
| HQ676238 | MY20_Sw_A_2006 | 2006 | B5 | E145Q | [10] |
| FM201323 | EV71_MY1764454_2006 | 2006 | B5 | - | [9] |
| HM358812 | EV0408_Penang_2008 | 2008 | B5 | E145G | [11] |
| HM358831 | EV0338_Sabah_2008 | 2008 | B5 | E98K | [11] |
| HM358810 | EV0336_Sabah_2008 | 2008 | B5 | E98K | [11] |
| HM358823 | EV0911_Kedah_2008 | 2008 | B5 | - | [11] |
| HM358818 | EV0764_Johor_2008 | 2008 | B5 | - | [11] |
| HM358816 | EV0577_Pahang_2008 | 2008 | B5 | - | [11] |
| HM358822 | EV0891_Johor_2008 | 2008 | B5 | - | [11] |
| HM358813 | EV0466_Johor_2008 | 2008 | B5 | - | [11] |
| HM358815 | EV0562_Penang_2008 | 2008 | B5 | - | [11] |
| HM358828 | EV1035_Pahang_2008 | 2008 | B5 | - | [11] |
| HM358824 | EV0943_Johor_2008 | 2008 | B5 | - | [11] |
| HM358830 | EV1094_Johor_2008 | 2008 | B5 | - | [11] |
| HM358819 | EV0811_Penang_2008 | 2008 | B5 | - | [11] |
| HM358825 | EV0972_Johor_2008 | 2008 | B5 | - | [11] |
| HQ676264 | MY47_Sw_A_2008 | 2008 | B5 | - | [10] |
| HM358827 | EV1025_Penang_2008 | 2008 | B5 | - | [11] |
| JN316096 | PM_2219140_2008 | 2008 | B5 | - | [3] |
| HM358817 | EV0758_Sabah_2008 | 2008 | B5 | - | [11] |
| HM358820 | EV0879_Bintulu_2008 | 2008 | B5 | - | [11] |
| HM358811 | EV0372_Sabah_2008 | 2008 | B5 | - | [11] |
| HM358829 | EV1078_Johor_2008 | 2008 | B5 | - | [11] |
| HM358821 | EV0884_Johor_2008 | 2008 | B5 | - | [11] |
| HM358809 | EV1075_Pahang_2008 | 2008 | B5 | - | [11] |
| HM358826 | EV1019_Penang_2008 | 2008 | B5 | - | [11] |
| HM358814 | EV0482_Sabah_2008 | 2008 | B5 | - | [11] |
| HQ676265 | MY48_Sw_A_2008 | 2008 | B5 | E145G | [10] |
| HM358833 | EV0076_KLumpur_2009 | 2009 | B5 | E145G | [11] |
| HM358832 | EV0031_Johor_2009 | 2009 | B5 | E145G | [11] |
| HM358835 | EV1945_Kuching_2009 | 2009 | B5 | - | [11] |
| HM358834 | EV1705_Johor_2009 | 2009 | B5 | E98K | [11] |
| KC894881 | EV1389-KLumpur_2010 | 2010 | B5 | - | [12] |
| KC894880 | EV1312-Johor_2010 | 2010 | B5 | - | [12] |
| KC894879 | EV1301-Melaka_2010 | 2010 | B5 | - | [12] |
| KC894878 | EV1299-Melaka_2010 | 2010 | B5 | - | [12] |
| KC894877 | EV1297-Melaka_2010 | 2010 | B5 | - | [12] |
| KC894876 | EV1233-Kedah_2010 | 2010 | B5 | - | [12] |
| KC894872 | EV0691-Terengganu_2010 | 2010 | B5 | - | [12] |
| KC894875 | EV0994-Terengganu_2010 | 2010 | B5 | - | [12] |
| KC894873 | EV0733-PPinang_2010 | 2010 | B5 | E145G | [12] |
| KC894874 | EV0744-Johor_2010 | 2010 | B5 | - | [12] |
| KC894866 | EV1056-Terengganu_2011 | 2011 | B5 | - | [12] |
| KC894869 | EV0984-Sarawak_2011 | 2011 | B5 | - | [12] |
| KC894867 | EV1268-Pahang_2011 | 2011 | B5 | - | [12] |
| KC894868 | EV0978-Sarawak_2011 | 2011 | B5 | - | [12] |
| KC894865 | EV1004-Terengganu_2011 | 2011 | B5 | - | [12] |
| KC894903 | EV0997-Pahang_2012 | 2012 | B5 | - | [12] |
| KC894902 | EV1325-Johor_2012 | 2012 | B5 | - | [12] |
| KC894899 | EV1002-Johor_2012 | 2012 | B5 | - | [12] |
| KC894894 | EV0891-Johor_2012 | 2012 | B5 | - | [12] |
| KC894883 | EV0616-Johor_2012 | 2012 | B5 | - | [12] |
| KC894882 | EV0615-Johor_2012 | 2012 | B5 | - | [12] |
| KC894900 | EV1003-Johor_2012 | 2012 | B5 | - | [12] |
| KC894887 | EV0673-Johor_2012 | 2012 | B5 | - | [12] |
| KC894884 | EV0655-Kedah_2012 | 2012 | B5 | - | [12] |
| KC894889 | EV0769-Johor_2012 | 2012 | B5 | - | [12] |
| KC894886 | EV0665-Kelantan_2012 | 2012 | B5 | - | [12] |
| KC894888 | EV0710-Johor_2012 | 2012 | B5 | - | [12] |
| KC894885 | EV0659-Pahang_2012 | 2012 | B5 | - | [12] |
| KC894901 | EV1170-Selangor_2012 | 2012 | B5 | - | [12] |
| KC894898 | EV0961-Johor_2012 | 2012 | B5 | - | [12] |
| KC894890 | EV0775-Johor_2012 | 2012 | B5 | - | [12] |
| KC894895 | EV0894-Kedah_2012 | 2012 | B5 | - | [12] |
| KC894896 | EV0896-Johor_2012 | 2012 | B5 | - | [12] |
| KC894897 | EV0953-Johor_2012 | 2012 | B5 | - | [12] |
| KC894891 | EV0779-Johor_2012 | 2012 | B5 | - | [12] |
| KC894893 | EV0834-Johor_2012 | 2012 | B5 | - | [12] |
| KC894892 | EV0791-Johor_2012 | 2012 | B5 | - | [12] |
| U22521 | BrCr_1969 | 1969 | A | NA |  |
| NA not applicable | |  |  |  |  |

References and bibliographic details

1. Unpublished, Brown,BA, Oberste,SM. and Pallansch,MA. Molecular epidemiology of enterovirus 71 isolates from fatal and nonfatal cases associated with a large outbreak of hand foot and mouth disease in Malaysia 1997.
2. Herrero LJ, Lee CSM, Hurrelbrink RJ, Chua BH, Chua KB, McMinn PC. Molecular epidemiology of enterovirus 71 in peninsular Malaysia, 1997-2000. Arch Virol. 2003; 148: 1369-1385.
3. Chan YF, Wee KL, Chiam CW, Khor CS, Chan SY, Amalina WMZ, et al. Comparative genetic analysis of VP4, VP1 and 3D gene regions of enterovirus 71 and coxsackievirus A16 circulating in Malaysia between 1997-2008. Trop Biomed. 2012; 29: 451-466.
4. Chan YF, AbuBakar S. Phylogenetic evidence for inter-typic recombination in the emergence of human enterovirus 71 subgenotypes. BMC Microbiol. 2006; 6: 74.
5. McMinn P, Lindsay K, Perera D, Chan HM, Chan KW, Cardosa MJ. Phylogenetic analysis of enterovirus 71 strains isolated during linked epidemics in Malaysia, Singapore, and Western Australia. J Virol. 2001; 75: 7732.
6. Yamayoshi S, Koike S. Identification of a human SCARB2 region that is important for enterovirus 71 binding and infection. J Virol. 2011; 85: 4937-4946.
7. Podin Y, Gias ELM, Ong F, Leong YW, Yew SF, Yusof MA, et al. Sentinel surveillance for human enterovirus 71 in Sarawak, Malaysia: lessons from the first 7 years. BMC Public Health. 2007; 6: 180.
8. Ooi MH, Wong SC, Podin Y, Akin W, del Sel S, Mohan A, et al. Human enterovirus 71 disease in Sarawak, Malaysia: A prospective clinical, virological, and molecular epidemiology study. Clin Infect Dis. 2007; 44: 646–656.
9. AbuBakar S, Sam IC, Yusof J, Lim MK, Misbah S, MatRahim N, et al. Enterovirus 71 outbreak, Brunei. Emerg Infect Dis. 2009; 15: 79-82.
10. McWilliam Leitch EC, Cabrerizo M, Cardosa J, Harvala H, Ivanova OE, Koike S, et al. The association of recombination events in the founding and emergence of subgenogroup evolutionary lineages of human enterovirus 71. J Virol. 2012; 86: 2676-2685.
11. Apandi MY, Fazilah R, Maizatul AA, Liyana AZ, Hariyati MA, Fauziah MK, et al. Molecular epidemiology of human enterovirus71 (HEV71) strains isolated in Peninsular Malaysia and Sabah from year 2001 to 2009. J Gen Mol Virol. 2011; 3: 18-26.
12. Yusof MA, Haryati MA, Hamadah MS, Noor KR, Zarina MZ, Syarifah NA, et al. Subgenogroup B5 maintains its supremacy over other human Enterovirus71 strains that circulated in Malaysia from 2010 to 2012. J Gen Mol Virol. 2014; 6: 1-5.
