# Supplementary material for: Cyclical Patterns of Hand, Foot and Mouth Disease Caused by Enterovirus A71 in Malaysia
Source: PLoS Negl Trop Dis. 2016 Mar 24;10(3):e0004562. doi: 10.1371/journal.pntd.0004562 (PMC4806993; doi:10.1371/journal.pntd.0004562)
Supplement: S3 Table — (DOCX) [file pntd.0004562.s003.docx]

**S3 Table: Seroprevalence rates of EV-A71 neutralizing antibody in children, 1995-2012**

| Year | Age group (%) | | |  |
| --- | --- | --- | --- | --- |
|  | 1-6 years | 7-12 years | Total | *P* value^a^ |
| 1995 | 54.0 (27/50) | 75.5 (40/53) | 65.0 (67/103) | 0.025 |
| 1996 | 47.8 (22/46) | 68.4 (52/76) | 60.7 (74/122) | 0.035 |
| 1997 | 51.5 (34/66) | 78.9 (56/71) | 66.2 (90/137) | 0.001* |
| 1998 | 55.2 (16/29) | 61.1 (22/36) | 58.5 (38/65) | 0.801 |
| 1999 | 55.0 (33/60) | 65.0 (13/20) | 57.5 (46/80) | 0.602 |
| 2000 | 79.1 (53/67) | 71.4 (35/49) | 75.9 (88/116) | 0.384 |
| 2001 | 42.3 (22/52) | 64.3 (27/42) | 52.1 (49/94) | 0.040 |
| 2002 | 64.8 (35/54) | 79.3 (23/29) | 69.9 (58/83) | 0.214 |
| 2003 | 57.1 (40/70) | 72.7 (32/44) | 63.2 (72/114) | 0.113 |
| 2004 | 53.8 (64/119) | 74.1 (60/81) | 62.0 (124/200) | 0.005 |
| 2005 | 28.7 (25/87) | 58.0 (40/69) | 41.7 (65/156) | <0.001* |
| 2006 | 36.1 (22/61) | 85.0 (17/20) | 48.2 (39/81) | <0.001* |
| 2007 | 45.0 (27/60) | 74.2 (23/31) | 54.9(50/91) | 0.014 |
| 2008 | 65.3 (49/75) | 78.9 (41/52) | 70.9 (90/127) | 0.115 |
| 2009 | 72.2 (13/18) | 91.7 (11/12) | 80.0 (24/30) | 0.358 |
| 2010 | 42.9 (18/42) | 70.6 (12/17) | 50.9 (30/59) | 0.084 |
| 2011 | 44.2 (19/43) | 56.3 (9/16) | 53.8 (28/59) | 0.559 |
| 2012 | 77.1 (27/35) | 76.5 (13/17) | 76.9 (40/52) | 1.000 |
| TOTAL | 52.8 (546/1034) | 71.6 (526/735) | 60.6 (1072/1769) | <0.001 |

^a^Fisher’s exact test was used to test the associations between age group and seroprevalence.

* *P*<0.05 after Bonferroni correction of type 1 family wise error accounting for multiple comparisons across individual years.
